# Pharmaceutical wastewater irrigation and metabolite-based environmental pharmacognosy: a perspective on quality and safety risks for medicinal plants

**DOI:** 10.3389/fphar.2026.1878168

**Published:** 2026-07-23

**Authors:** Edith O. Diovu, Charles O. Nnadi, Ugwu Okechukwu Paul-Chima

**Affiliations:** 1 Department of Pharmacognosy and Environmental Medicine, Faculty of Pharmaceutical Sciences, University of Nigeria Nsukka, Nsukka, Enugu, Nigeria; 2 Department of Pharmaceutical and Medicinal Chemistry, Faculty of Pharmaceutical Sciences, University of Nigeria Nsukka, Nsukka, Enugu, Nigeria; 3 Department of Research and Publication, Kampala International University, Kampala, Uganda

**Keywords:** botanical-drug safety, contaminants of emerging concern, environmental pharmacognosy, medicinal plants, metabolite reprogramming, metabolomics, pharmaceutical wastewater

## Abstract

Medicinal plants are increasingly cultivated in agroecosystems irrigated with treated or untreated wastewater, biosolids and contaminated surface water. Pharmaceutical residues are recognised contaminants of emerging concern, but their implications for botanical-drug quality, therapeutic consistency and safety remain insufficiently characterised. This Perspective argues that pharmaceutical wastewater irrigation is a plausible yet underexamined driver of metabolite reprogramming in medicinal plants. Chronic exposure to antibiotics, non-steroidal anti-inflammatory drugs, antiepileptics, antidepressants, hormones and transformation products may alter secondary metabolism through oxidative stress, xenobiotic detoxification, rhizosphere microbiome disturbance and modified nutrient signalling. These processes may change phenolic, flavonoid, alkaloid, terpenoid, glycoside and volatile metabolites that underpin pharmacognostic quality and ethnopharmacological reliability. Medicinal plants may also accumulate parent pharmaceuticals, transformation products and, under some conditions, microbial signatures associated with antibiotic resistance. Building on Carter et al.’s source-pathway-receptor framework and Helmecke et al.’s regulatory risk synthesis, we shift attention from residue burden to how exposure history alters the medicinal metabolome. Evidence from antibiotic-induced metabolite changes in *Pinellia ternata* supports this proposition, while indicating compound- and context-specific effects. We advance a balanced position: metabolite reprogramming is biologically credible, but food-crop studies often report de minimis residue risks and inconsistent rhizosphere-resistome effects. Future work should integrate wastewater profiling, matched controls, targeted and untargeted metabolomics, transformation-product discovery, microbiome analysis, digestion and bioaccessibility testing, bioactivity assays and probabilistic mixture-risk assessment.

## 1 Introduction

Medicinal plants remain central to primary healthcare, nutraceutical development and pharmaceutical discovery. Their therapeutic value depends not only on botanical identity but also on the qualitative and quantitative integrity of secondary metabolites. Conventional pharmacognostic assessment therefore emphasises authentication, macroscopic and microscopic characterisation, marker-metabolite quantification, microbial contamination analysis, pesticide residue testing, heavy metal profiling and adulteration detection. This framework remains essential, but it is incomplete where medicinal plants are cultivated in agroecosystems exposed to complex anthropogenic contaminant mixtures (Gworek et al., 2021; Mosharaf et al., 2024).

**FIGURE 1:**
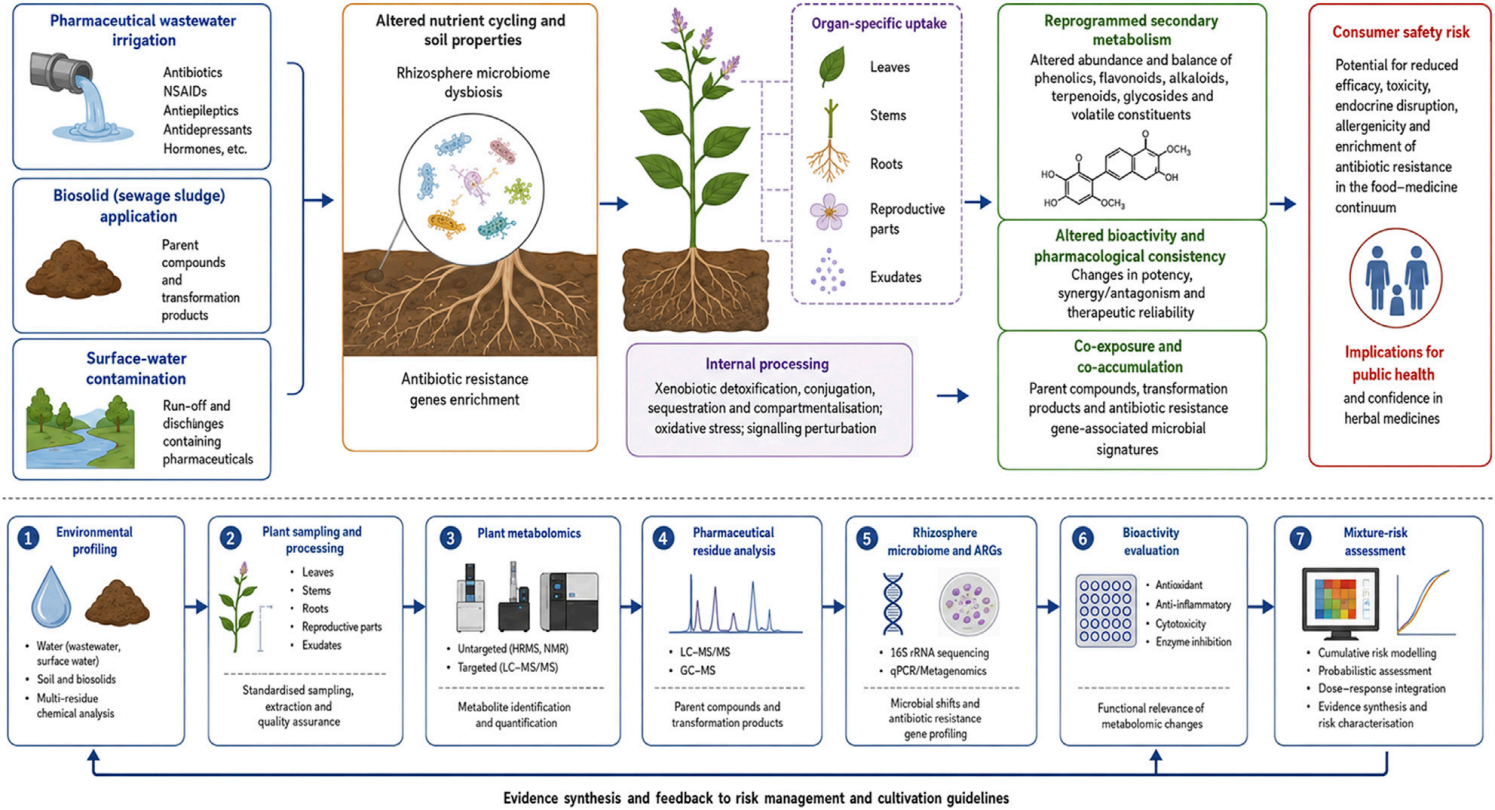
Conceptual exposure-to-quality framework for environmental pharmacognosy.

Pharmaceutical residues are a consequential class of contaminants of emerging concern because they are biologically active by design, continuously discharged and often incompletely removed during wastewater treatment (Carter et al., 2019; Gworek et al., 2021; Helmecke et al., 2020). Reclaimed wastewater, untreated effluent, sewage sludge and biosolids may introduce antibiotics, analgesics, antiepileptics, antidepressants, hormones and other pharmaceuticals into agricultural soils (Table 1; Figure 1) (Boxall et al., 2006; Carter et al., 2014; Kumar et al., 2005). Plant uptake has been demonstrated in leafy vegetables, root crops and cereals (Boxall et al., 2006; Carter et al., 2014; Miller et al., 2016; Tanoue et al., 2012; Wu et al., 2012). Medicinal plants require a distinct analytical framework because they are consumed specifically for bioactive chemistry and are judged by pharmacognostic consistency rather than agronomic yield alone.

**TABLE 1:** Pharmaceutical compound classes relevant to wastewater irrigation and their potential pharmacognostic implications.

Pharmaceutical class	Representative compounds	Plant-uptake evidence	Pathways most likely affected	Pharmacognostic implication
Antibiotics	Tetracyclines, sulfonamides, fluoroquinolones	Uptake documented in roots, leaves and edible tissues across crop systems (Boxall et al., 2006; Carter et al., 2014; Kumar et al., 2005; Tanoue et al., 2012); direct medicinal-plant evidence now available (Duan et al., 2025)	Nitrogen metabolism; alkaloid biosynthesis; rhizosphere microbial diversity	Possible alteration of alkaloid potency; potential, but context-dependent, enrichment of antibiotic-resistance genes in harvested material (Christou et al., 2017; Gatica and Cytryn, 2013; Han et al., 2022)
Non-steroidal anti-inflammatory drugs	Ibuprofen, diclofenac, naproxen, paracetamol	Root and shoot detection with translocation to leaf tissue in model systems (Bartha et al., 2010; Carter et al., 2014; Miller et al., 2016)	Phenylpropanoid and flavonoid biosynthesis; reactive-oxygen-species homeostasis; salicylate-dependent signalling	Possible dysregulation of anti-inflammatory phenolic metabolites and competition between xenobiotic conjugation and endogenous glycoside formation
Antiepileptic and psychoactive compounds	Carbamazepine, lamotrigine, fluoxetine	Carbamazepine is persistent and mobile in soil-plant systems (Carter et al., 2014; Miller et al., 2016; Wu et al., 2012)	Membrane transporters; terpenoid and volatile biosynthesis; stress-response networks	Potential shifts in essential-oil composition and volatile metabolite fingerprints
Hormones and endocrine-active substances	Oestradiol, ethinylestradiol, testosterone, bisphenol A	Uptake reported in root and vegetative tissues (Adeel et al., 2017; Carter et al., 2014)	Phytoestrogen and flavonoid biosynthesis; auxin and cytokinin signalling	Possible disruption of phytoestrogenic or adaptogenic metabolite profiles
Antihypertensive and cardiac-active compounds	Atenolol, metoprolol, enalapril, digoxin	Emerging evidence of uptake in leafy species, with limited direct evidence in medicinal plants (Carter et al., 2014; Mosharaf et al., 2024)	Carbon and nitrogen allocation; stress-responsive phenolic and terpenoid flux	Theoretical interaction between trace residues and plant metabolites sharing cardiovascular targets

Evidence is drawn mainly from food-crop and model-plant systems, with the recent exception of Duan et al. (2025) in a medicinal species. The predominance of food-crop and model-system evidence reflects the structure of research funding and scientific priority rather than evidence of absence; reasons are discussed in Section 3.1. ARG, antibiotic-resistance gene; GC-MS, gas chromatography-mass spectrometry; LC-MS/MS, liquid chromatography-tandem mass spectrometry; NSAIDs, non-steroidal anti-inflammatory drugs.

The central concern is not simply whether medicinal plants accumulate pharmaceutical residues. The more important question is whether chronic wastewater exposure reprogrammes the plant metabolome in ways that compromise therapeutic quality, pharmacological reliability and consumer safety. A medicinal plant may remain taxonomically authentic while its metabolite profile is environmentally distorted. This possibility is a recognised gap in botanical-drug quality control and provides the rationale for a metabolite-based environmental pharmacognosy framework.

### 1.1 Positioning against prior wastewater-irrigation frameworks

Two bodies of work define the current conceptual landscape and must be acknowledged explicitly. Carter et al. (2019) applied source-pathway-receptor analysis to develop a holistic framework for evaluating the impacts of pharmaceuticals present in wastewater used for irrigation on agroecosystems and human health, identifying a large number of processes and compartments for which usable data were largely absent. Helmecke et al. (2020) examined the regulatory dimension of agricultural water reuse and the risks posed by organic micro-contaminants, arguing that existing reuse guidelines inadequately address pharmaceuticals. These frameworks are foundational, and the present Perspective does not seek to displace them.

Environmental pharmacognosy extends the source-pathway-receptor logic in one specific direction that those frameworks did not develop. In the Carter et al. (2019) scheme the plant and the human consumer are receptors at which a residue concentration is evaluated against a toxicological threshold. We propose that, for medicinal plants, the plant is not only a receptor of the contaminant but also a biochemical respondent whose therapeutic value is itself an endpoint. The pathway of concern therefore does not end at residue accumulation; it continues into the secondary metabolism that constitutes the medicinal product. In this reframing the receptor of ultimate interest is the consumer of a botanical drug whose pharmacological profile may have been altered, with or without measurable parent-residue accumulation. Environmental pharmacognosy thus adds a metabolite-integrity endpoint to the source-pathway-receptor structure rather than proposing a competing structure, and it specifies the analytical instrumentation, namely, targeted and untargeted metabolomics, required to make that endpoint measurable.

The framework links pharmaceutical wastewater exposure, biosolid application and surface-water contamination to soil and rhizosphere disturbance, organ-specific plant uptake, xenobiotic detoxification, stress-mediated secondary-metabolite flux, altered bioactivity and consumer-safety risk. The analytical workflow integrates water and soil profiling, targeted and untargeted plant metabolomics, in planta transformation-product discovery, pharmaceutical residue quantification, rhizosphere microbiome and antibiotic-resistance-gene analysis, *in vitro* digestion and bioaccessibility testing, bioactivity assays and probabilistic mixture-risk assessment (Table 1; Figure 1).

## 2 Pharmaceutical wastewater as a hidden pharmacognostic stressor

Pharmaceutical wastewater is a dynamic exposure matrix whose composition varies with domestic medicine use, hospital discharge, manufacturing effluent, seasonal hydrology and treatment-plant efficiency. Even when individual compounds occur at low concentrations, medicinal plants may experience prolonged multi-compound exposure throughout germination, vegetative growth, flowering and the developmental windows in which medicinally relevant metabolites accumulate.

Such exposure may not produce visible phytotoxicity. Instead, it may induce subtle biochemical responses detectable through metabolomics, transcriptomics or pathway-level chemical profiling. Pharmaceutical residues may elevate reactive oxygen species, alter antioxidant defence systems, interfere with phytohormone signalling, affect membrane-transporter activity, disturb nitrogen and carbon allocation and activate xenobiotic detoxification pathways (Adeel et al., 2017; Bartha et al., 2010; Carter et al., 2014). Because many medicinally important secondary metabolites are stress responsive, including phenylpropanoids, alkaloids and terpenoids, these exposures may increase, suppress or qualitatively reshape metabolite production. This proposition is no longer purely inferential: Duan et al. (2025) reported that oxytetracycline, norfloxacin and sulfamethazine produced distinct, compound-specific shifts in growth, redox homeostasis and secondary metabolism in the medicinal species Pinellia ternata (Thunb.) Makino (Araceae), with oxytetracycline promoting and sulfamethazine suppressing the accumulation of particular metabolite classes.

A central methodological caution follows directly from the nature of wastewater. Wastewater differs from clean irrigation water not only in its pharmaceutical content but also in nutrient load, salinity, dissolved organic matter and microbial inoculum. Any of these can alter the metabolome independently of the pharmaceutical fraction. A metabolite difference observed between wastewater-irrigated and clean-water-irrigated plants therefore cannot, by itself, be attributed to pharmaceuticals. We address this confounding explicitly in the study design proposed in Section 7.

A medicinal plant cultivated under wastewater irrigation may therefore satisfy morphological and genetic identity criteria while showing altered concentrations of key bioactive metabolites. This concern is particularly important for species whose traditional or clinical use depends on a narrow metabolite balance, essential-oil composition or standardised extract profile. The ecological provenance of cultivation water should therefore be treated as a pharmacognostic variable rather than as a peripheral agricultural detail.

## 3 Metabolite reprogramming and therapeutic inconsistency

Plant secondary metabolism is plastic. Phenylpropanoid, flavonoid, alkaloid, terpenoid and glycoside pathways respond to environmental stress, microbial interactions and xenobiotic exposure (Bartha et al., 2010; Carter et al., 2014; Gworek et al., 2021). Pharmaceutical residues may therefore act as cryptic ecological signals that redirect biosynthetic flux without causing obvious morphological change.

Phenolic and flavonoid metabolites may increase as part of oxidative-stress defence, yet specific metabolites required for anti-inflammatory or antioxidant activity may decrease. Hormone residues could alter phytoestrogenic or adaptogenic profiles (Adeel et al., 2017). Alkaloid biosynthesis, which depends on nitrogen metabolism and stress-signalling intermediates, may be perturbed by altered soil nitrogen dynamics. Terpenoid and volatile metabolite profiles may shift through changes in plastidial and mevalonate pathway activity, with consequences for antimicrobial efficacy, pharmacopoeial identity and commercial value. Glycosylation and conjugation pathways may also be modified because plants use these mechanisms to detoxify xenobiotics and sequester reactive intermediates (Carter et al., 2014; Tanoue et al., 2012). The Pinellia ternata data of Duan et al. (2025) provide a concrete illustration that the direction of change is not uniform but depends on the specific antibiotic, which cautions against any assumption that wastewater exposure produces a single predictable metabolite signature.

Single-marker assays are insufficient to detect this complexity. An extract may contain an acceptable level of one pharmacopoeial marker while showing substantial disruption of associated metabolites that contribute to synergy, solubility or bioavailability. Targeted quantification of recognised markers should therefore be combined with untargeted metabolomics to distinguish environmentally induced reprogramming from ordinary concentration variability.

### 3.1 Why direct evidence in medicinal plants remains limited

Until recently, the evidence for pharmaceutical uptake and metabolite reprogramming was drawn almost entirely from food crops and model species, particularly Brassica juncea (L.) Czern. (Brassicaceae), Lactuca sativa L. (Asteraceae) and *Triticum aestivum* L. (Poaceae). The study by Duan et al. (2025) in Pinellia ternata is an important early exception that moves part of the argument from inference to direct observation. Even so, direct experimental evidence across the diversity of pharmacopoeially important species remains sparse, and this reflects structural constraints rather than a lack of plausibility.

Several factors account for this gap. First, research on crop uptake of pharmaceutical residues has been funded primarily in the context of food safety and human dietary exposure, where high-yield staple and vegetable species are natural study subjects. Medicinal plants occupy a scientifically valuable but commercially marginal niche that has attracted comparatively limited dedicated funding in environmental pharmacology. Second, medicinal-plant secondary metabolomes are substantially more complex than those of model species. This chemical richness makes it technically more demanding to distinguish pharmaceutical-induced metabolomic shifts from natural genotypic, seasonal and post-harvest variation. Third, pharmacognostic research and environmental contaminant science have operated as largely separate communities, with few methodological frameworks bridging the two domains. The environmental pharmacognosy framework proposed here is intended to address this structural disconnect. Directed investment in controlled exposure studies using pharmacopoeially important species, such as those proposed in Section 7, is required to move the field from plausible inference to broad experimental validation.

## 4 The rhizosphere microbiome as a mechanistic mediator

The rhizosphere microbiome links wastewater exposure to medicinal-plant chemistry. Root-associated bacteria and fungi regulate nutrient acquisition, phytohormone balance, induced systemic resistance and secondary-metabolite elicitation. Antibiotics and disinfectant compounds in irrigation water may, under some conditions, reduce beneficial microbial diversity, select resistant taxa and impair pathways involved in nitrogen cycling, phosphate solubilisation and plant-defence priming (Christou et al., 2017; Han et al., 2022; Mosharaf et al., 2024).

This points to a three-stage mechanism in which pharmaceutical residues disturb the soil and rhizosphere environment, altered microbial communities modify plant-microbe signalling, and the plant metabolome changes through indirect biological pathways. Where it occurs, this microbiome-mediated effect may be as important as direct residue uptake.

The strength and even the direction of these microbiome effects are, however, contested, and an honest account must say so. Gatica and Cytryn (2013) found that irrigation with treated municipal wastewater did not measurably increase antibiotic-resistance levels in the soil microbiome, concluding that the high background resistance in both treated-wastewater-irrigated and freshwater-irrigated soils largely reflected native soil populations rather than wastewater input. A subsequent systematic review of treated and untreated wastewater irrigation likewise concluded that effects on soil and water antimicrobial resistance are inconsistent and strongly dependent on water quality, soil type, climate and study design (Slobodiuk et al., 2021). Several field studies have similarly reported limited impact of water type on rhizosphere community composition. The enrichment of antibiotic-resistance genes and the disruption of beneficial taxa should therefore be presented as possible and context-dependent outcomes requiring site-specific verification, not as predictable consequences of wastewater use. This uncertainty is itself an argument for the integrated, control-matched study design proposed below, because only such designs can establish when microbiome-mediated effects are real and material.

A robust research design must therefore examine irrigation water, bulk soil, rhizosphere, root endosphere, aerial organs and final botanical-drug material as connected compartments within a single exposure-to-risk continuum. Treating medicinal plants as isolated metabolite containers obscures the mechanisms that connect environmental contamination to pharmacognostic outcome.

## 5 Safety considerations beyond conventional quality control

Botanical-drug safety assessment traditionally prioritises heavy metals, pesticides, microbial load, mycotoxins, adulterants and botanical substitution. Pharmaceutical residues are rarely incorporated into routine medicinal-plant quality standards despite their biological activity, environmental persistence and potential for chronic low-level consumer exposure.

Exposed medicinal plants may present overlapping issues. Parent pharmaceuticals may accumulate in roots, leaves, flowers, seeds or bark depending on polarity, ionisation, persistence and plant physiology. Biotransformation products generated through plant metabolism and soil microbial activity may differ toxicologically from the parent compound. Antibiotic residues and metabolites may, in some settings, continue selecting for resistance determinants in the rhizosphere, on plant surfaces and during storage. Botanical preparations already contain multiple pharmacologically active metabolites that may modify the absorption, distribution or elimination of trace residues in consumers.

This account must, however, be balanced against the food-crop risk-assessment literature. Several field studies of staple and vegetable crops irrigated with treated wastewater have estimated that human dietary exposure to individual pharmaceutical residues falls well below toxicological thresholds of concern, supporting a de minimis characterisation of single-compound risk under typical treated-wastewater conditions (Carter et al., 2019; Mosharaf et al., 2024; Prosser and Sibley, 2015). We do not dispute these findings. The argument advanced here is narrower and complementary in three respects. First, those assessments evaluate residue toxicity to the consumer, whereas the principal concern for medicinal plants is the alteration of the therapeutic metabolome, which a residue-threshold calculation does not capture. Second, medicinal plants are consumed deliberately as concentrated extracts and over prolonged periods for specific pharmacological effect, a pattern of intake that differs from incidental dietary exposure. Third, even where individual residues fall below thresholds, concurrent exposure to structurally or pharmacologically related plant metabolites complicates mixture-risk assessment.

A medicinal plant used for inflammation, hypertension, anxiety or glycaemic dysregulation could theoretically contain trace pharmaceuticals acting on overlapping biological targets. This does not imply inevitable clinical harm, and the balance of current evidence does not support alarm about parent-residue toxicity. It does, however, justify systematic residue screening, metabolite-integrity assessment, mixture-risk evaluation and declaration of cultivation-water quality within Good Agricultural and Collection Practice guidance (World Health Organization, 2003).

## 6 Environmental pharmacognosy: proposed framework

Environmental pharmacognosy should connect the ecological exposure history of a medicinal plant to the integrity of its secondary metabolome and the safety of derived preparations. Its premise is that medicinal-plant quality cannot be fully assessed without characterising the environmental conditions under which the metabolome was produced. This framework does not replace conventional authentication; it extends it with an ecological dimension that is directly relevant to ethnopharmacological quality, and it operationalises the metabolite-integrity endpoint that distinguishes it from the residue-focused receptor of earlier source-pathway-receptor models (Carter et al., 2019; Helmecke et al., 2020).

A study implementing this framework should begin with chemical profiling of irrigation water and bulk soil for priority pharmaceuticals and transformation products. Botanical identity should be confirmed using voucher specimens, morphology and DNA barcoding, with full species names, authorities and families validated against the Medicinal Plant Names Services and Plants of the World Online. Plant tissues should be analysed using targeted and untargeted LC-MS/MS metabolomics, while volatile-rich species should also undergo GC-MS profiling, with metabolite identification and reporting following Metabolomics Standards Initiative conventions (Sumner et al., 2007). In planta transformation-product discovery using high-resolution and ion-mobility mass spectrometry should be incorporated, because plant and microbial biotransformation can generate metabolites that are absent from the irrigation water yet relevant to consumer safety. Pharmaceutical residues should be quantified separately in roots, stems, leaves and the medicinally used organ to establish uptake and translocation coefficients. Rhizosphere microbiome profiling using 16S rRNA sequencing or shotgun metagenomics, combined with antibiotic-resistance-gene panel analysis, should clarify whether microbial disturbance mediates metabolite change or constitutes an independent safety pathway, while recognising the context-dependence discussed in Section 4. Bioactivity assays should test whether metabolomic differences translate into altered anti-inflammatory, antimicrobial, antioxidant or other validated functions, and *in vitro* digestion and bioaccessibility testing should establish what fraction of any residue or altered metabolite is released under physiologically relevant conditions.

This approach would classify medicinal-plant material into four quality categories: authentic plants with stable, exposure-unperturbed metabolomes; authentic plants with environmentally altered metabolite profiles; authentic plants containing measurable pharmaceutical residues without appreciable metabolomic change; and plants showing combined metabolite reprogramming and direct contamination. This classification is more informative than a binary exposed-versus-control comparison and is better suited to risk-stratified regulatory decisions.

## 7 Research priorities

A high-level investigation should use controlled greenhouse or field exposure models. Crucially, and in direct response to the confounding problem identified in Section 2, the design must include nutrient-matched and salt-matched controls so that pharmaceutical-specific effects can be disentangled from those caused by the nutrient, salinity and organic-matter content of wastewater. A defensible factorial design would compare, at minimum, clean water; conventionally treated wastewater; untreated or minimally treated wastewater; a synthetic nutrient-and-salinity matrix reconstructed to match the wastewater but free of pharmaceuticals; and that same matrix spiked with a defined synthetic pharmaceutical mixture at environmentally relevant concentrations. Comparison of the pharmaceutical-free matched matrix with its spiked counterpart isolates the pharmaceutical contribution, while comparison of the matched matrix with clean water quantifies the nutrient and salinity contribution. Without this nutrient-matched arm, metabolomic differences cannot be causally assigned to pharmaceuticals.

Candidate species could include Moringa oleifera Lam. (Moringaceae), Ocimum gratissimum L. (Lamiaceae), Artemisia annua L. (Asteraceae), Mentha × piperita L. (Lamiaceae) and Azadirachta indica A. Juss. (Meliaceae), which combine public-health relevance, recognised pharmacopoeial markers and existing pharmacological validation. All species nomenclature has been validated taxonomically, and full authorities and families are given at first mention throughout.

The primary outcome should be metabolomic alteration in the medicinally used organ, analysed with multivariate chemometrics and pathway enrichment. Secondary outcomes should include pharmaceutical residue concentrations, organ-specific translocation factors, transformation products identified by high-resolution and ion-mobility mass spectrometry, rhizosphere microbiome diversity, antibiotic-resistance-gene abundance, bioactivity-assay results, bioaccessible fractions from *in vitro* digestion and probabilistic consumer-risk estimates. Multi-centre and multi-season studies are needed because wastewater composition and plant metabolomes vary with geography, season, genotype, developmental stage, harvest timing and post-harvest handling.

### 7.1 Chemometric and machine-learning strategies for environmental biomarker discovery

The complexity of untargeted LC-MS/MS and GC-MS datasets generated from wastewater-exposed medicinal plants poses substantial analytical challenges. Datasets in this domain are typically high-dimensional, comprising thousands of features per sample, and are subject to considerable biological variation arising from genotype, harvest stage, season and geography. Conventional multivariate approaches, including principal component analysis, partial least-squares discriminant analysis and hierarchical cluster analysis, provide important dimensionality reduction and pattern-recognition capability. These methods may be supplemented by supervised machine-learning algorithms that offer superior discriminatory power when the goal is to identify true environmental biomarkers rather than to characterise overall metabolomic structure.

In experimental scenarios where the number of observations exceeds the number of features, ensemble tree-based methods such as random forests, gradient-boosting frameworks including XGBoost and CatBoost, and support vector machines with appropriate kernels are particularly well suited to this task. Random forests provide non-parametric feature-importance rankings that are robust to multicollinearity, a common property of closely related metabolite signals. XGBoost and CatBoost extend this capability with iterative residual fitting that tends to reduce overfitting when applied to structured tabular data derived from these complex matrices. Regularised regression approaches such as elastic net can further assist variable selection when the dataset is sparse or when feature reduction prior to pathway enrichment is required.

A practical analytical workflow might proceed in three stages. In the first stage, raw metabolomic data would undergo preprocessing including peak picking, alignment, normalisation and missing-value imputation. In the second stage, supervised machine-learning models trained on exposure-group labels would identify candidate biomarker features, with permutation testing used to assess significance and cross-validation or independent test sets used to estimate generalisation performance. In the third stage, shortlisted features would enter pathway-enrichment analysis using curated databases such as KEGG, PlantCyc or HMDB. This tiered approach balances the complementary strengths of statistical metabolomics and data-driven machine learning and is consistent with emerging best practice in environmental metabolomics and exposomics. Throughout, metabolite annotation confidence should be reported against Metabolomics Standards Initiative levels (Sumner et al., 2007) so that biomarker claims remain auditable.

Regulatory translation should focus on environmental quality markers for Good Agricultural and Collection Practice guidance (World Health Organization, 2003), including contaminant thresholds in irrigation water, metabolomic deviation indices relative to curated reference metabolomes, antibiotic-resistance-gene abundance cut-offs where context warrants, and minimum reporting standards for cultivation-water quality. These measures would make ecological exposure history auditable rather than anecdotal.

### 7.2 Practical feasibility: academic research versus routine pharmacopoeial implementation

The analytical workflow described in Figure 1 is intentionally comprehensive, designed to establish the scientific foundations of environmental pharmacognosy rather than to prescribe a routine quality-control procedure. It is therefore important to distinguish two implementation contexts: exploratory academic research and routine pharmacopoeial or regulatory application.

In an academic or capacity-building context, the full workflow is realistic at established research institutions with access to high-resolution mass spectrometry, sequencing infrastructure and bioinformatics capability. A phased approach is recommended, in which initial studies prioritise wastewater chemical profiling, targeted residue quantification and untargeted metabolomics, before adding rhizosphere metagenomics, transformation-product discovery and probabilistic risk assessment as the evidence base grows. International collaborations and shared facilities can reduce the per-study cost of metagenomics and high-throughput metabolomics, and platforms such as the Global Natural Products Social Molecular Networking infrastructure support open metabolomics data sharing.

For routine application in resource-limited pharmacopoeial settings, the full workflow is not currently feasible and is not proposed as such. A tiered pragmatic model is more appropriate. At the first tier, cultivation-water quality documentation, basic soil-residue screening and targeted quantification of priority pharmaceuticals using validated LC-MS/MS methods could be incorporated into Good Agricultural and Collection Practice audits at relatively modest cost. At the second tier, a subset of biomarker metabolites identified from full academic-grade studies could be integrated into simplified marker panels for routine quality assessment. At the third tier, probabilistic mixture-risk assessment and metagenomics would remain the domain of specialised reference laboratories supporting regulatory decision-making. This tiered structure parallels the model already used in pesticide-residue monitoring and heavy-metal profiling for botanical materials, where complexity is stratified by regulatory purpose rather than applied uniformly.

## 8 Discussion and contribution to the field

This Perspective reframes pharmaceutical wastewater as both a contamination source and a potential driver of change in therapeutic chemistry. Existing pharmacognostic frameworks centre on identity, marker metabolites, microbial contamination, heavy metals and pesticide residues. They do not adequately address the possibility that chronic low-dose pharmaceutical exposure may reprogramme the secondary metabolome on which medicinal value depends. Relative to the source-pathway-receptor frameworks of Carter et al. (2019) and Helmecke et al. (2020), the specific contribution here is to define metabolite integrity as a distinct receptor-level endpoint for medicinal plants and to specify the metabolomic instrumentation required to measure it.

The framework strengthens the fit between environmental science and ethnopharmacology by treating ecological provenance as a determinant of medicinal-plant quality. For plant materials used in traditional medicine, therapeutic meaning is inseparable from the chemical integrity of the harvested organ and the reproducibility of bioactive profiles. Environmental pharmacognosy therefore provides a practical route for evaluating whether the conditions of cultivation have preserved or altered the metabolite architecture that supports ethnopharmacological use. The recent demonstration of antibiotic-induced, compound-specific metabolite shifts in Pinellia ternata (Duan et al., 2025) shows that this is an empirical and tractable question rather than a speculative one, while the inconsistent resistome literature (Gatica and Cytryn, 2013; Slobodiuk et al., 2021) and the reassuring food-crop residue-risk findings (Prosser and Sibley, 2015) jointly caution against overstatement.

By integrating wastewater chemistry, plant metabolomics, transformation-product discovery, rhizosphere metagenomics, residue quantification, bioaccessibility testing, bioactivity assays and mixture-risk assessment, environmental pharmacognosy offers a pathway for expanding botanical-drug safety standards to include exposure history, metabolome integrity and consumer safety. This is particularly relevant to Frontiers in Pharmacology because it links environmental exposure, pharmacological quality and the safety of biologically active plant preparations within a single translational framework.

## 9 Conclusion

Pharmaceutical wastewater irrigation is an emerging and insufficiently recognised consideration for medicinal-plant quality and safety. Its significance extends beyond contaminant accumulation because chronic environmental exposure may reprogramme the secondary metabolism that underpins therapeutic value. A medicinal plant may be botanically authentic yet pharmacognostically altered by its exposure history, a distinction that current quality-control frameworks are not equipped to detect, and one now supported by direct evidence in at least one medicinal species. At the same time, the available food-crop risk evidence and the inconsistent resistome literature indicate that the appropriate posture is rigorous investigation rather than alarm. Metabolite-based environmental pharmacognosy offers a scientifically defensible, experimentally feasible and regulatorily translatable framework for identifying these alterations, linking them to mechanisms, distinguishing pharmaceutical effects from matrix effects through matched controls, and incorporating verified findings into improved botanical-drug safety standards. Environmental exposure history should become an auditable dimension of medicinal-plant quality assessment, supported by standardised metabolomics reporting, harmonised residue panels and risk-proportionate pharmacopoeial guidance.

## Data Availability

The original contributions presented in the study are included in the article/supplementary material, further inquiries can be directed to the corresponding author.
